# Cell Membrane- and Extracellular Vesicle-Coated Chitosan Methacrylate-Tripolyphosphate Nanoparticles for RNA Delivery

**DOI:** 10.3390/ijms252413724

**Published:** 2024-12-23

**Authors:** Wen Jie Melvin Liew, Syed Abdullah Alkaff, Sheng Yuan Leong, Marin Zhen Lin Yee, Han Wei Hou, Bertrand Czarny

**Affiliations:** 1 School of Materials Science and Engineering, Nanyang Technological University, Singapore 639798, Singapore; 2 School of Mechanical and Aerospace Engineering, Nanyang Technological University, Singapore 639798, Singapore; 3 Lee Kong Chian School of Medicine, Nanyang Technological University, Singapore 308232, Singapore

**Keywords:** nanolipogel, extracellular vesicles, drug delivery system, membrane coating, RNA

## Abstract

mRNA-based vaccines against the COVID-19 pandemic have propelled the use of nucleic acids for drug delivery. Conventional lipid-based carriers, such as liposomes and nanolipogels, effectively encapsulate and deliver RNA but are hindered by issues such as premature burst release and immunogenicity. To address these challenges, cell membrane-coated nanoparticles offer a promising alternative. We developed a novel nanoparticle system using chitosan methacrylate-tripolyphosphate (CMATPP), which capitalizes on interactions involving membrane proteins at biointerfaces. Ionic crosslinking between chitosan methacrylate and tripolyphosphate facilitates the formation of nanoparticles amenable to coating with red blood cell (RBC) membranes, extracellular vesicles (EVs), and cell-derived nanovesicles (CDNs). Coating CMATPP nanoparticles with RBC membranes effectively mitigated the initial burst release of encapsulated small interfering RNA (siRNA), sustaining controlled release while preserving membrane proteins. This concept was extended to EVs, where CMATPP nanoparticles and CDNs were incorporated into a microfluidic device and subjected to electroporation to create hybrid CDN-CMATPP nanoparticles. Our findings demonstrate that CMATPP nanoparticles are a robust siRNA delivery system with suppressed burst release and enhanced membrane properties conferred by cell or vesicle membranes. Furthermore, the adaptation of the CDN-CMATPP nanoparticle formation in a microfluidic device suggests its potential for personalized therapies using diverse cell sources and increased throughput via automation. This study underscores the versatility and efficacy of CMATPP nanoparticles in RNA delivery, offering a pathway towards advanced therapeutic strategies that utilize biomimetic principles and microfluidic technologies.

## 1. Introduction

**Figure 1:**
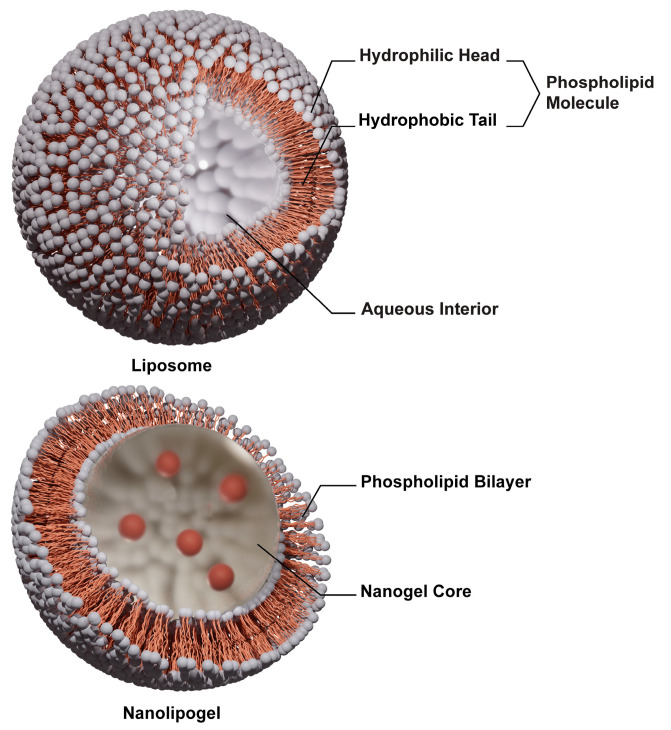
Structure of a liposome and nanolipogel.

Nucleic acid delivery has gained significant traction in biomedical research, particularly since the development of mRNA-based vaccines against the COVID-19 pandemic. However, premature burst release is a major problem in the delivery of hydrophilic biomolecules, such as antibodies, peptides, siRNA, and mRNA, which may lead to a higher dosage frequency for the desired therapeutic effect [1]. Hence, controlled and sustained release is often preferred to avoid systemic and liver toxicity associated with higher dosages, particularly under chronic conditions [2]. Liposomes are among the most widely researched methods of delivery for hydrophilic molecules owing to their flexibility and versatility in design and fabrication to improve their biological properties, such as biocompatibility and systemic circulation [3]. They can hold both hydrophobic molecules within the bilayer as well as hydrophilic molecules in the aqueous core [4]. However, they still have limited effectiveness in controlling the burst release of hydrophilic cargo as well as immunogenicity issues [5], leading to the development of nanolipogels (NLGs), where there is an additional nanogel core, depicted in Figure 1 below, as a strategy for controlling the burst release of encapsulated cargo [6,7,8]. Previous studies have shown that NLGs have been successfully used to encapsulate and suppress the burst release of hydrophilic drugs and biomolecules, such as doxorubicin hydrochloride [9], dextran-FITC (DFITC) [5,10], maraviroc, and tenofovir disoproxil fumarate [6]. However, these studies used synthetic lipids such as egg phosphatidylcholine (EPC), distearoylphosphatidyl ethanolamine (DSPE), and 1,2-dioleoyl-3-trimethylammonium ropane (DOTAP) [6,9,11] for gel encapsulation, which can potentially lead to immunoreactions.

**Figure 2:**
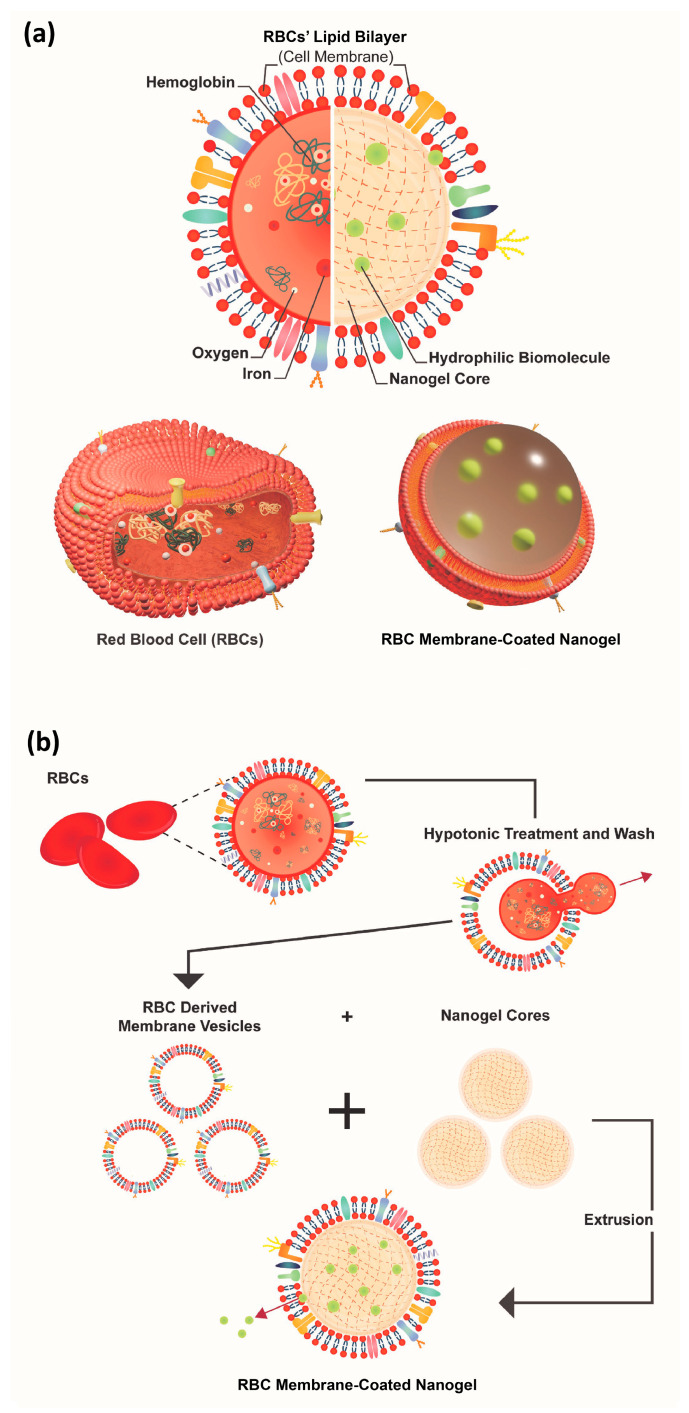
Overall schematic of the (**a**) comparison between RBC and RBC membrane-coated nanogel (RBC-NG) and (**b**) the formation of RBC-NG. Harvested RBC membrane vesicles are co-extruded with nanogels to form RBC-NGs, which retain the surface markers and membrane proteins from the RBC membranes.

On the other hand, cell membrane coatings show potential for additional benefits, especially for biological interactions, which largely involve membrane proteins on particle biointerfaces [12]. In particular, cell and biomimicry are emerging as promising areas of research that leverage the interactions and mechanisms crafted by nature through evolutions [13,14,15], such as ghost cell membranes that can be used as a membrane coating for biomimetic NLG systems. The cell types explored include platelets [16], white blood cells [17], cancer cells [18], stem cells [19], and endothelial cells [20]. Among these, RBCs have emerged as a forerunner in the choice of cell membrane due to their natural long circulation and lifetime (120 days in humans) and low immune cell clearance [12]. In addition, RBCs’ membranes can reassemble or reseal into vesicles after hemolysis and coat nanoparticles if present during the resealing process [21]. In fact, nanogels coated with RBC membranes were shown to retain membrane proteins and complexity while having a nanogel core, as shown in Figure 2a. They have also been shown to have higher survival rates than free molecules and do not elicit anti-RBC antibodies even after repeated dosages [22], nor PEG IgG and IgM antibodies, and hence have significant performance in prolonged circulation, consistent over multiple administrations by avoiding accelerated blood clearance (ABC) [12]. These proved to be more immunogenic than the PEGylated nanoparticles.

In devising the harvesting process, since membrane proteins make up about 52% of the membrane mass, with lipids making up 40% and 8% carbohydrate [23], it is important to extract the RBC membrane as a whole rather than synthetically conjugating the components onto a lipid bilayer [24]. RBC-coated nanoparticles have been shown to successfully translocate membrane proteins, such as the self-marker CD47, with a density similar to that of the source RBC and on the right-side-out orientation [25], which is critical for proper functionalization. Moreover, RBC membrane-coated nanoparticles have a greater suppressed release [19,26], signifying that the RBC membrane coating can provide a barrier against the diffusion of encapsulated molecules. The RBC membrane-coated nanogel with a core-shell structure is shown in Figure 2b.

Similarly, cell-derived nanovesicles (CDNs) [27] and extracellular vesicles (EVs), such as exosomes, can be used as membrane coatings for nanoparticles due to their specific intracellular and surface membrane proteins [28,29], which can provide higher systemic circulation and organotrophic homing behavior [29]. These proteins include CD47 [30], an integrin-associated transmembrane protein that initiates the signal that inhibits phagocytosis [31], CD55, and CD59, and glycosylphosphatidylinositol (GPI)—linked complement regulatory proteins that work in tandem to provide protection against complement attacks [32], thereby increasing the stability of exosomes in circulation. In addition, exosome membranes contain tetraspanin CD9 and CD81, which promote membrane fusion with cells [33], allowing exosome vesicles to facilitate intercellular delivery. These EVs are even found to be able to cross and penetrate the dense tissue structure of the blood–brain barrier [34,35], signifying its potential for drug delivery applications.

Most studies involving coated nanogel cores have used synthetic polymers such as poly(lactic acid) (PLA) [26], poly(lactic-co-glycolic acid) (PLGA) [36,37] and polycaprolactone (PCL) [38]. They are commonly used because of their biocompatibility, stability, and ease of fabrication. However, for in vivo applications, they undergo acidic degradation by-products [39], limiting their range of applications. Similarly, delivery studies involving exosome membrane-coated nanoparticles mostly used PLGA and gold nanomaterials, such as nanorods and nanocages [35,40,41]. As such, there is a need for the discovery of a more favorable nanogel core for drug delivery with similar biocompatibility and biodegradable properties, yet with non-acidic degradation by-products. Ideally, the crosslinking density and size should also have a certain degree of controllability, with an increased affinity for biomolecules. Similarly, batch-to-batch differences and labor-intensive processes hinder the effectiveness of EV coating [41], leading to the exploration of alternative fabrication methods. Microfluidic systems have emerged as a novel method for coating cell membranes onto nanoparticles via rapid mixing on a microfluidic chip [42]. Microfluidic systems offer the advantages of high throughput, parallel dependence, and quantitative format [43], making them an attractive area of exploration for cell membrane coating techniques. Microfluidics can be produced using microfabrication techniques to integrate different functionalities and components, allowing for the efficient and precise control of the microenvironment of the process [44]. Electroporation is one such function. Electroporation has been widely used for cell transfection, where applied electric fields break down the dielectric layer over cell membranes, causing the transient formation of pores for biomolecules to enter [45]. However, increasing temperatures during electroporation may be a concern, and the throughput is limited [46]. Therefore, this presents an opportunity to combine electroporation with microfluidic technology for a microfluidic electroporation system, which can enhance throughput and flow process control. Cell membranes or EVs can then be introduced into the microfluidic chip together with nanoparticles in separate inlets and passed through an electroporation zone. The applied electric field can then induce pore formation in EVs or opening of the membrane, allowing the nanoparticles to enter [42], resulting in cell membrane-coated nanoparticles.

**Figure 3:**
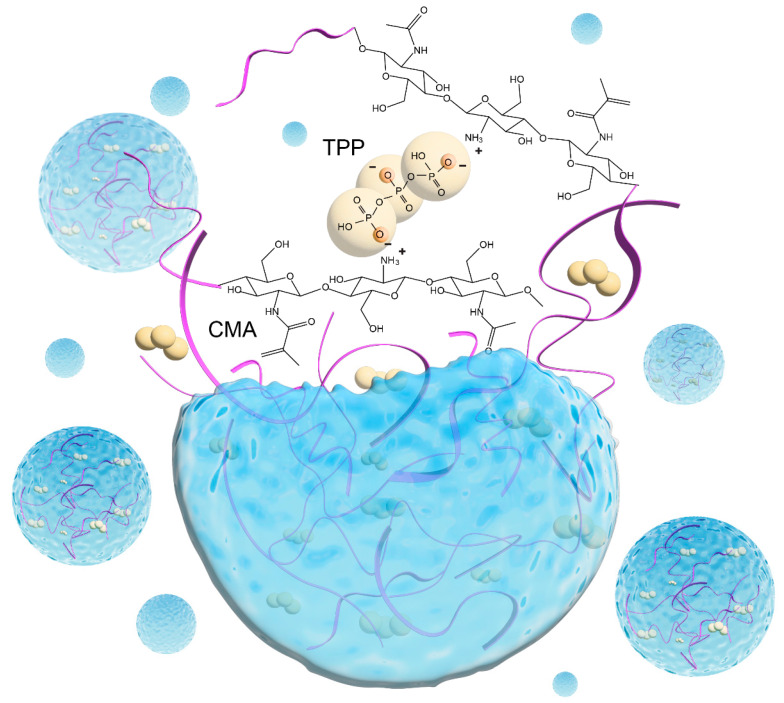
Schematic of ionic crosslinking between anionic TPP and cationic ends of CMA in CMATPP nanoparticles.

Therefore, in this study, we developed a novel nanoparticle system using chitosan methacrylate (CMA) for ionic gelation with sodium tripolyphosphate (TPP) to allow coating with synthetic lipids and cell membranes. As shown in Figure 3, the polyanion of TPP acts as an ionic crosslinker between the two cationic ends of the neighboring CMA chains, forming CMATPP nanoparticles.

Chitosan has been previously shown to form nanoparticles using the ionic gelation method with TPP [47,48]. However, ionic gelation in chitosan-TPP nanoparticles is susceptible to changes in pH, such as incomplete solubilization of chitosan at pH above 5 [49] and competition of ionic linkage with the amino groups of chitosan between OH ions and phosphoric ions at higher pH [50]. The ionic crosslink between chitosan and TPP is also pH responsive [51] and debonds or uncrosslinks at physiological pH, bringing about the risk of rapid cargo release.

Using CMA to ionically crosslink with TPP confers the fabricated nanoparticles with the ability to be photocrosslinked under UV light to form covalent crosslink points that are not susceptible to pH changes, allowing for application at physiological pH and better control of the encapsulation and suppression of the initial burst release of biomolecules. After ionic gelation, the CMATPP nanoparticles were further crosslinked by exposure to UV light, creating covalent crosslinks in the MA conjugates. With changes in pH, only ionic crosslinks are susceptible to debonding, whereas covalent crosslinks are more stable. These covalently crosslinked nanoparticles allow co-extrusion with cell membrane vesicles to form cell membrane-coated CMATPP NLGs, which are stable at physiological pH and do not undergo acidic degradation by-products, as opposed to commonly used nanogel cores for cell membrane coating, such as PLA, PLGA, and PCL, as mentioned previously.

Previous research has shown that particle formation, hydrodynamic diameters, and polydispersity are affected by the mass ratio of chitosan to TPP, as well as the stirring speed during fabrication [52,53]. Building on this, we developed a set of parameters that have the best repeatability and size range for our applications, such as membrane coating. The CMATPP nanoparticles also allow for coating with synthetic lipid bilayers, such as Egg PC, and cell-derived membranes, such as RBC vesicles and CDNs. The coating was performed in four ways: thin-film hydration with EPC, co-extrusion with RBC ghost vesicles or U937 CDNs, and microfluidic electroporation with CDNs.

In this study, methacrylate was conjugated onto chitosan to form CMA, which was ionically crosslinked with TPP to form CMATPP nanoparticles that were able to undergo subsequent photocrosslinking to form a CMATPP nanogel. The nanogel particles were then used for co-extrusion with both RBC and CDN to form RBC membrane-coated RBC-CMATPP NLGs and CDN membrane-coated CDN-CMATPP NLGs. A novel electroporation microfluidic system was used as an alternative to co-extrusion, as a proof of concept for an alternate fabrication system. Flow cytometry and zeta potential measurements were used to confirm successful coating. Small interfering RNA (siRNA) was then encapsulated and its release was studied over a 14-day period.

## 2. Results

### 2.1. CMATPP NLG Characterization and Coating

The ^1^H NMR spectrum was used to characterize the chemical structure of the CMA, as shown in Figure S1 in Supplementary Materials. Vinyl protons are present at 5.57 and 5.78 ppm (g, 2H, CH_2_) and methyl protons of methacrylic anhydride residues are present at 1.88 ppm (h, 3H, CH_3_). Such unique chemical shift peaks for vinyl protons at 5.5 and 6.0 ppm were not present in the native chitosan [54,55], reflecting successful MA conjugation onto chitosan to form CMA. The SD% of the modified CMA was calculated to be 21.9% from the ^1^H NMR spectrum, confirming successful conjugation between methacrylic anhydride and the amino groups on chitosan.

Meanwhile, as seen from the hydrodynamic size data presented in Table 1, CMATPP nanoparticles and all coated CMATPP NLGs displayed similar hydrodynamic sizes of approximately 200 nm, with a PDI of approximately 0.3 and below, indicating that the methodology can produce homogenous nanoparticles with a narrow size distribution. However, it should be noted that the hydrodynamic sizes of samples with cellular membranes, such as RBC ghosts, RBC CMATPP, U937 CDNs, and U937 CDN CMATPP, tended to be larger than those of samples with synthetic phospholipids, such as EPC bare liposomes and EPC CMATPP. Although the EPC lipid bilayer and RBC membrane were measured to be approximately 5 nm [56,57], RBC membranes have a much more complex composition, consisting of approximately 52% proteins and 40% lipids, including phospholipids and cholesterol [23]. This means that RBC membranes have less fluidity than EPC liposomes and that membrane proteins may contribute to the measured hydrodynamic diameter. This is in line with research data showing that coating with RBC membranes results in an approximately 20 nm increase in hydrodynamic diameter [15,26].

**Figure 4:**
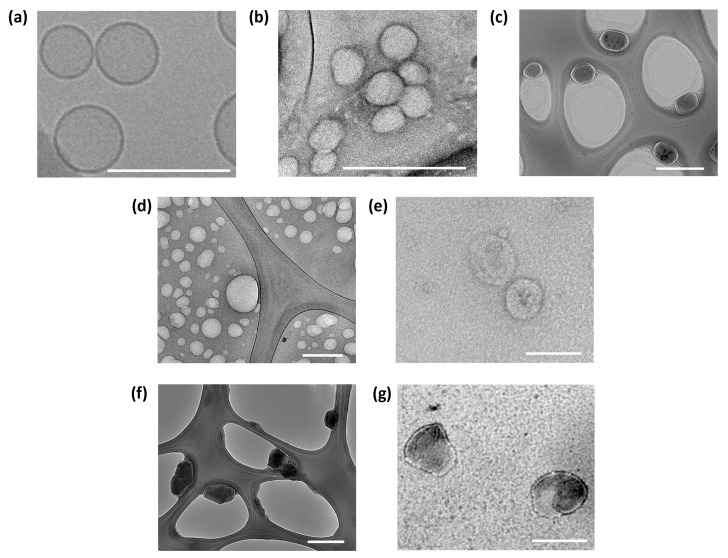
TEM images for (**a**) bare EPC liposomes (cryogenic), (**b**) CMATPP nanoparticles, (**c**) EPC CMATPP NLGs, (**d**) RBC ghost vesicles, (**e**) RBC CMATPP NLGs, (**f**) CDNs, (**g**) CDN CMATPP NLGs. Images are taken with uranyl acetate staining. Unmarked scale bars in the figures represent 200 nm.

In addition, the zeta potential data, which reflect the charges of the particles, coated EPC CMATPP, RBC CMATPP, and CDN CMATPP NLGs, showed a decrease in the zeta potential from the highly positive CMATPP nanoparticles. This implies that the CMATPP nanoparticles were successfully coated on all the coating vesicles, including EPC liposomes, RBC ghost vesicles, and CDNs, as all had a negative zeta potential. The zeta potential graph, Figure S2 in the Supplementary Materials, also shows a single peak distribution, reflecting the complete coating of all the CMATPP particles. TEM imaging was used to observe the morphologies of the fabricated CMATPP NLGs while cryogenic TEM was used to observe the morphology of bare EPC liposomes, Figure 4a, to prevent the inevitable collapse of the lipid bilayer. Looking at Figure 4b,c, CMATPP nanoparticles and EPC CMATPP NLGs were both slightly smaller than 200 nm and spherical in shape, considering the possible slight deformations from the drying process during preparation for imaging. Similarly, when looking at RBC ghost vesicles in Figure 4d, RBC ghost vesicles of spherical shapes and nanosizes can be obtained from the developed harvest procedure and after coating of CMATPP particles, RBC CMATPP in Figure 4e showed similar morphology and sizes. These observations also hold true for CDN in Figure 4f and CDN CMATPP in Figure 4g. Taken together, these results show that the thin-film hydration and co-extrusion methods can produce homogenously coated CMATPP NLGs consistently, consistent with the DLS size distribution data.

**Figure 5:**
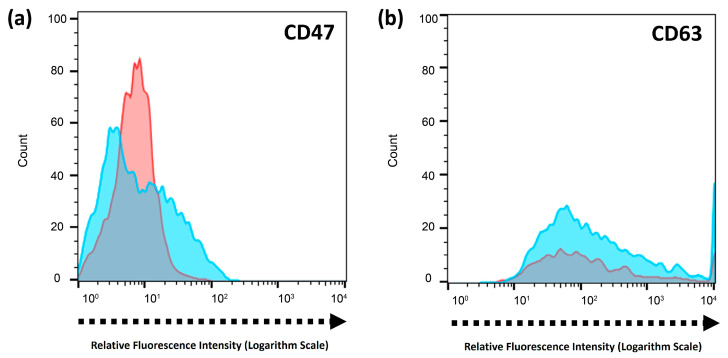
(**a**) Flow cytometry of RBC self-marker CD47’s distribution in RBC ghost vesicles (red line) and co-extruded RBC CMATPP NLGs (blue line); (**b**) flow cytometry of CDN surface protein marker’s (Tetraspanin CD63) distribution in CDNs (red line) and co-extruded CDN CMATPP NLGs (blue line).

Flow cytometry was performed to check for the presence of coating via the preservation of surface protein markers after co-extrusion. CD47 is a self-marker on the RBC membrane that prevents elimination by macrophages by interacting with signal regulatory protein alpha (SIRPα) on macrophages and prolonging blood circulation [12,58]. The presence of a coating can be deduced by comparing CD47 on the RBC and CMATPP NLGs. Figure 5a shows that CD47 was present on RBC and RBC CMATPP NLGs, indicating the presence of an RBC membrane coating on the CMATPP nanoparticles. This is supported by the zeta potential data in Table 1, which show a change in zeta potential from a positive charge for CMATPP nanoparticles to a negative charge for RBC CMATPP, which can only be achieved by coating with negatively charged RBC ghost membranes. Similarly, CD63, a surface protein of CDN, can be seen in Figure 5b was expressed by both CDNs and CDN CMATPP NLGs. This strongly suggests the successful coating of CDN onto CMATPP. This coincides with the zeta potential data, which shows a drop after coating, similar to the RBC CMATPP NLGs.

### 2.2. In Vitro Release Studies

**Figure 6:**
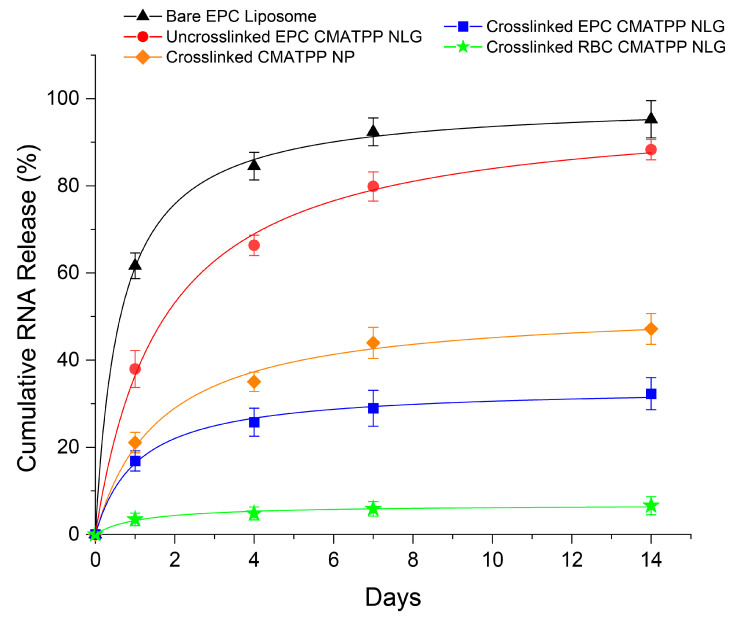
In vitro release profiles for siRNA encapsulated in bare liposome (black, triangle), uncrosslinked EPC CMATPP NLG (red, circle), crosslinked CMATPP nanogel (brown, diamond), crosslinked EPC CMATPP NLG (blue, square), and crosslinked RBC CMATPP NLG (green, star). (*n* = 3) Error bars represent the SD of the values.

EPC CMATPP and RBC CMATPP were used to encapsulate and release siRNA to test their potential as drug delivery systems for charged biomolecules. The release profiles are shown in Figure 6. Thin-film hydration was used to coat the siRNA-CMATPP nanoparticles with an EPC lipid bilayer. Coated EPC CMATPP NLGs were then photocrosslinked to study the release profile of crosslinked EPC CMATPP NLGs, uncrosslinked EPC CMATPP NLGs, and bare liposomes. In addition, co-extrusion was performed with CMATPP nanoparticles and RBC ghost vesicles to form coated RBC CMATPP NLGs. CMATPP nanoparticles were photocrosslinked and resuspended in a non-acidic buffer prior to co-extrusion with RBC vesicles to preserve membrane proteins and markers from RBC membranes. The drug release profiles of siRNA from RBC CMATPP NLGs were studied and also presented in Figure 6.

The release of siRNA from CMATPP NLGs was monitored for 14 days under physiological conditions. As shown in Figure 6, uncrosslinked and crosslinked EPC CMATPP NLGs can suppress the initial burst release, with up to a 3-fold and 2-fold difference for crosslinked and uncrosslinked EPC CMA NLGs, respectively, compared to bare EPC liposomes. Although uncrosslinked EPC CMATPP NLGs lack a nanogel core because they are not photocrosslinked, some degree of suppression is still present in the ionic network from the fabrication of the CMATPP core, providing some resistance to the diffusion of the encapsulated siRNA out of the particles [5]. In contrast, crosslinked EPC CMATPP NLGs have a covalently crosslinked nanogel core, effectively hindering the diffusion of encapsulated siRNA to a much greater degree. Under physiological conditions and pH, CMA loses its charge, and ionic crosslinking is compromised. The covalent crosslinks present in the crosslinked CMATPP NLGs remained intact to hold the polymeric network together. This will allow the continuous suppression of burst release and sustained release of encapsulated siRNA in crosslinked CMATPP NLGs. In addition, the release of siRNA was also slowed to a greater extent by 30% for crosslinked EPC CMATPP NLGs and by up to 80% over 14 days for uncrosslinked EPC CMATPP NLG. From these results, it is reasonable to deduce that EPC CMATPP NLGs can effectively suppress the initial burst release of siRNA. Furthermore, the coating of CMATPP nanoparticles could be changed to RBC vesicles, which showed near-complete suppression of siRNA release, as shown in Figure 6. The nanogel core in RBC CMATPP NLGs is expected to have a similar degree of suppression of the initial burst release and sustained release as crosslinked EPC CMATPP NLGs, as their nanogel cores are chemically identical. However, the difference in coating caused further suppression of the initial burst release and sustained release, indicating that RBC membrane coating provides higher drug retention by acting as a diffusional barrier [26]. Furthermore, siRNA has a strong anionic charge, which may result in electrostatic repulsion from the anionic RBC membrane coating [59], similar to the impermeability of cell membranes [60]. In addition, referring to the hydrodynamic size data in Table 1, it is also possible that RBC CMATPP NLGs have a multilayer membrane coating compared to EPC CMATPP NLGs, which will present additional diffusional barriers to the encapsulated siRNA. This could also be expected for CDN CMATPP NLGs, as they are both coated with membranes of cellular origin.

Taken together, CMATPP NLGs are suitable for the encapsulation and sustained release of charged biomolecules such as siRNA. However, the length of RNA can influence the release profile, as it alters the N/P ratio between CMATPP and RNA. A higher N/P ratio results in more uncomplexed CMA sites, which may increase crosslinking points with TPP. This leads to a higher crosslinking density, thereby suppressing RNA release. For the encapsulation of other drugs, charge is the primary factor influencing the release profile, assuming a molecular weight similar to that of siRNA described in this study. Negatively charged drugs are likely to exhibit enhanced encapsulation in the positively charged CMA system, resulting in a release profile comparable to that of siRNA. Conversely, neutral or uncharged drugs will not form ionic interactions with the nanogel. Their encapsulation and release will primarily depend on the nanogel’s mesh properties, as described in our previous study [5]. In the reported formulations, the release profile for uncharged drugs follows a similar trend but shows less pronounced suppression of the initial burst release. This is because the release is predominantly governed by the drug’s diffusion coefficient through the nanogel core and across the membrane.

The fabrication method also allows for different coatings of CMATPP nanoparticles to form CMATPP NLGs with different membrane coatings, which will give different degrees of drug retention and release.

This opens up the possibility of using a variety of synthetic and natural membrane sources, such as EPC, Soy PC, DOTAP, RBC, and other cell types, to equip CMATPP NLGs with a variety of membrane properties, from diffusional barriers to stealth delivery and active targeting. The high retention ability of RBC CMATPP may also be extended to CDN-coated CDN CMATPP NLGs, owing to their similar cellular membrane origins.

### 2.3. Microfluidics Coating of CDN CMATPP NLG

A microfluidic system was used to coat the CDNs derived from U937 cells. Microfluidics is an emerging and novel method for coating nanoparticles, as it combines electroporation and mixing onto a single chip [42,43]. Given their highly automatable procedure, microfluidic systems can provide higher throughput than conventional lipid-coating methods [43].

**Figure 7:**
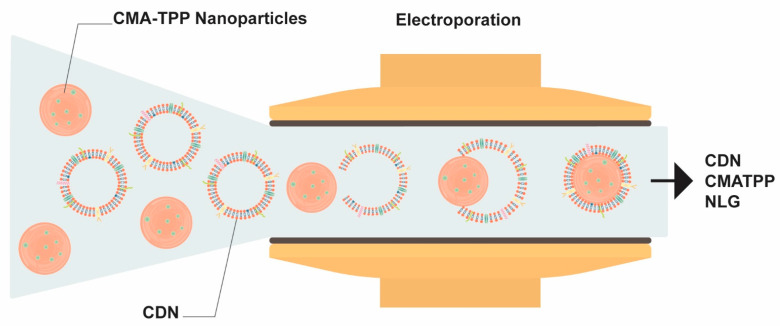
Overview of the channel in a microfluidic device for fabrication of coated CDN CMATPP NLGs, synthesized by passing through an electroporation zone under continuous flow.

CDNs and CMATPP nanoparticles were passed through the electroporation zone, where pores were formed on the CDNs’ membranes owing to the electric field. This enabled the loading of CMATPP to form CDN-coated CMATPP NLGs without any chemical modification of the membrane or CMATPP nanoparticles. The overall schematic is shown in Figure 7. The hydrodynamic sizes of the samples before and after microfluidic electroporation were measured and are presented in Table 2. Zeta potential analysis of the CDN CMATPP NLGs showed an overall negative charge, signifying the successful coating of negatively charged CDNs onto positively charged CMATPP particles. Particle concentration was also measured using nanoparticle tracking analysis, as presented in Table 2. The minimum CDN required was estimated using the surface area to be coated [61], where approximately three CDNs were required to fully coat two CMATPP particles. CDN was then used in excess to ensure efficient coating of all CMATPP nanoparticles. We also observed that electroporation at 0.8 V caused a decrease in particle concentration of approximately 15%, which suggests that there were interactions between the CMATPP and CDNs, as shown in Figure S3 in the Supplementary Materials. Flow cytometry was conducted to determine the presence of tetraspanins (CD9 and CD81) and multivesicular body (MVB) protein markers (TSG101) [27].

**Figure 8:**
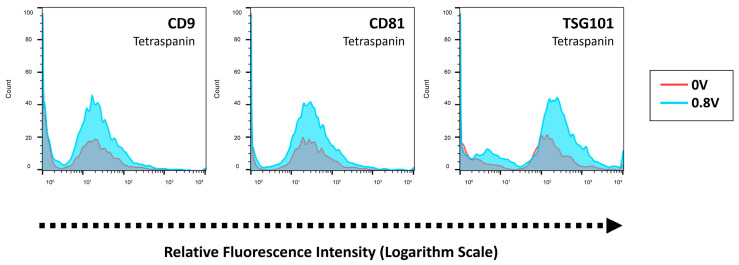
Flow cytometry of three CDN characteristic protein markers’ (tetraspanins: CD9 and CD81, MVB: TSG101) distribution in mixture of CDNs with CMATPP nanoparticles, electroporated under different conditions. Red line depicts electroporation performed at 0V, blue line depicts electroporation performed at low frequency (100 Hz, 0.8 V, 1 ms pulse width) and yellow line depicts electroporation performed at high frequency (1000 Hz, 2.5 V, 10 μs pulse width).

CDNs and CMATPP nanoparticles were individually passed through the electroporation zone at low and high frequencies to study their effects on particle integrity. Samples were perfused into the chip multiple times at different frequencies. Low-frequency electroporation was performed at 100 Hz, with a 0.8 V and 1 ms pulse width, and high-frequency electroporation was performed at 1000 Hz, with 2.5 V and 1μs. As shown in Figure S4 in the Supplementary Materials, CDNs were first tested at different frequencies, and the group that underwent high-frequency electroporation presented a lower intensity of the protein markers tested, indicating that the membrane integrity of the CDNs was compromised. In comparison, CDNs that underwent low-frequency electroporation had relatively similar intensities for all three proteins tested. However, looking at CMATPP’s corresponding flow cytometry data from Figure S5 in the Supplementary Materials, electroporation at a high frequency resulted in a higher intensity for protein detection. As there is only non-specific binding between CMATPP nanoparticles and proteins, the data suggest a possible disruption of the CMATPP nanoparticle structure, which opens up the available binding sites on the particles. As any compromise on particle or membrane integrity is undesirable, low-frequency electroporation was selected when both CDN and CMATPP nanoparticles were electroporated together to coat the CMATPP nanoparticles. This is also in line with the literature, which showed that low-frequency electroporation creates higher permeabilization in the electroporation of cells [62]. As shown in Figure 8, when passing a mixture containing CDNs and CMATPP nanoparticles through the electroporation zone, there were overlaps in signal intensity for all three proteins expressed, signifying that characteristic proteins were preserved after electroporation, indicating the successful coating of CDN onto CMATPP nanoparticles. This was also supported by the higher signal counts for all three protein expressions, which reflected a better coating of CDN onto the CMATPP nanoparticles. Taken together, microfluidics electroporation was successfully used to coat CDNs onto CMATPP nanoparticles, and this coating method can also be very versatile by using CDNs from different cell types.

## 3. Discussion

Nanogel cores encapsulated within a phospholipid bilayer, nanolipogels, are an approach to exert control over the initial burst release of hydrophilic cargo during their release.

In this study, we showed that methacrylate can be conjugated onto chitosan to fabricate CMA, and this functionalization allows chitosan to retain its biocompatibility [63] while having the ability to be photocrosslinked to secure the payload. Successful conjugation was observed in the NMR data, and it was further demonstrated that CMA could be ionically gelated with TPP to form CMATPP particles, which could be further processed into lipid CMATPP and membrane CMATPP nanoparticles for the encapsulation and delivery of biomolecules, such as siRNA. The method of fabricating CMATPP nanoparticles, separate from the formation of membrane vesicles, allows the co-extrusion of CMATPP nanoparticles and membrane vesicles for coating. This can endow NLG membranes with properties such as better immunogenicity, increased stealth against the immune response, reduced accelerated blood clearance, and slower transmembrane diffusion for better payload retention, all of which can be found in either exosomes or red blood cell membranes [12,22,30,31]. Common methods for membrane coating involve the co-extrusion of membrane vesicles with solid nanoparticles, which is essentially different from the one-pot fabrication method of thin-film hydration. Commonly used polymeric nanoparticles include PLA, PLGA, and PCL; however, they can potentially produce acidic degradation by-products [64], limiting their use. Therefore, our novel particle system was developed using ionic gelation between CMA and TPP to form CMATPP nanoparticles, which can be photocrosslinked prior to co-extrusion. This allows co-extrusion with membrane vesicles for the coating onto CMATPP nanoparticles. The methacrylate conjugates also allow photocrosslinking for the formation of a nanogel core that is stable in an uncharged environment. Crosslinked CMATPP nanoparticles have a positive surface charge, and their hydrodynamic size and zeta potential are shown in Figure S6 in the Supplementary Materials to be stable in PBS at pH 7.

With our robust fabrication process, the results showed that when co-extruded with RBC ghost vesicles, CMATPP nanoparticles can be coated to form RBC CMATPP NLGs with good repeatability and a smaller mean size for RBC CMATPP, presumably owing to the complexity of the RBC membrane. The successful coating of RBC CMATPP is also supported by a change in the zeta potential of the nanoparticles from positive CMATPP to negative CMATPP. This was achieved while retaining the spherical morphology of the nanoparticles, as shown in the TEM images in Figure 4. Nonetheless, for a more robust determination of the coating, flow cytometry was employed, which also showed the presence of CD47, a characteristic self-marker of RBCs, on the CMTAPP nanoparticles. Next, the ability of RBC CMATPP to encapsulate and sustain the release of biomolecules such as siRNA is evident. siRNA is a prominent approach to gene silencing [65], but it faces barriers during delivery, such as difficult entry into cells, stability issues, and intracellular transport [11]. Our approach can potentially solve this problem by introducing siRNA during the fabrication of CMATPP nanoparticles, which can be encapsulated into RBC CMATPP nanoparticles, which is then sustained release, with a suppression of the initial burst release, up to a 5-fold difference compared with cross-linked EPC CMATPP NLGs. Indeed, similar trends have been observed in previous studies where RBC-coated PLA nanoparticles showed a lower release rate of the encapsulated drug [26]. This observation can be extended to the fact that a simple phospholipid bilayer also acts as a barrier to the diffusion of the encapsulated drug [66]. This clearly indicates that the RBC membrane coating on CMATPP was able to elicit greater suppression of the burst release of the encapsulated biomolecules. In addition, RBC CMATPP NLGs contain membrane proteins preserved from RBC ghost vesicles, possibly inheriting longer circulation times and reduced clearance properties of RBC. In addition, CMATPP NLGs can also provide control over the encapsulation and release of siRNA, potentially allowing siRNA to be released only after NLGs are taken up by the target cells. To test with other cell lines, the membrane coating concept was then extended to other vesicles, such as CDNs, which can be considered as extracellular vesicles mimetics [27], which can be obtained from various cell sources. Co-extrusion with CDN showed similar results for zeta potential and hydrodynamic size data, which strongly suggests the success of the coating. Building on this, the CDN shows promise for application in other fabrication methods. One such method explored the application of microfluidic electroporation to coat the membrane onto CMATPP nanoparticles. The flow cytometry data in Figure 8 show that low-frequency electroporation induced the coating of CDN onto CMATPP nanoparticles to form CDN CMATPP NLGs, and specific markers of CDNs, CD9, CD81, and TSG101 were preserved throughout the coating process. This indicates the successful coating and probable retention of the membrane properties by the CDNs. Microfluidic electroporation opens up the potential for automation in the fabrication process and a higher throughput in commercialized processes.

Furthermore, EVs coating ideology can potentially be extended to other cell sources. Examples include the use of central nervous system cells for nanoparticle coating for targeted and enhanced uptake [67] and hybrid cellular membrane nanovesicles, and fusion of cellular vesicles from cancer cell lines for immunomodulation [68,69]. This opens a gateway for the fabrication of coated CMATPP nanoparticles with different membrane properties retained from different cell lines for advanced drug delivery applications, such as targeted delivery.

## 4. Materials and Methods

### 4.1. Chemicals and Reagents

Chitosan (50 kDa), acetic acid, methacrylic anhydride, sodium triphosphate pentabasic (TPP), lithium phenyl-2,4,6-trimethylbenzoylphosphinate (LAP), triton X-100, hydrochloric acid (HCl), sodium chloride (NaCl), and sodium azide were obtained from Merck (Singapore)). L-α-Phosphatidylcholine (egg, chicken) (EPC) was procured from Avanti Polar Lipids (Alabaster, AL, USA), and PELCO NetMesh™ TEM support grids were obtained from Ted Pella Inc (Redding, CA, USA). Nuclepore polycarbonate track-etched membranes were obtained from Fisher Scientific Pte Ltd (Singapore). Spectra/Por^®^ Biotech Cellulose Ester (CE) Membrane (1000 kDa) and Quant-it™ RiboGreen RNA Assay Kit were procured from Thermo Fisher Scientific (Singapore) Polydimethylsiloxane (PDMS) was procured from the Dow Chemical Company (Midland, MI, USA) and epoxy from MG Chemicals (Burlington, ON, Canada). All antibodies used in flow cytometry were procured from Santa Cruz Biotechnology Inc. (Dallas, TX, USA). siRNA was procured from Sangon Biotech (Shanghai) Co., Ltd., (Songjiang, Shanghai, China), with the sense sequence being AACAAGACCUUCGACUCUUCC and antisense sequence GGAAGAGUCGAAGGUCUUGUU.

Dulbecco’s modified Eagle’s medium (DMEM), Roswell Park Memorial Institute (RPMI)-1640 medium, 0.25% trypsin-EDTA, phosphate-buffered saline (PBS), heat-inactivated fetal bovine serum (FBS), and penicillin streptomycin (Pen Strep) were procured from Gibco, Life Technologies (Carlsbad, CA, USA).

The cell line and human monocyte U937 cells were purchased form ATCC^®^ and cultured in RPMI-1640 medium supplemented with 10% FBS and 1% Pen Strep. All cell cultures were maintained in 5% CO_2_ at 37 °C, and adherent cells were subcultured with 0.25% trypsin-EDTA when 80% confluence was reached in T175 cell culture flasks.

### 4.2. Fabrication of CMATPP NLGs

#### 4.2.1. Synthesis of CMA

Chitosan methacrylate (CMA) was fabricated via the methacrylation of chitosan. A total of 3 wt% was added to 2% acetic acid and dissolved overnight with constant stirring. 1 M of methacrylate anhydride per repeating unit of chitosan was added dropwise while the solution was stirred at 60 °C. After 6 h, the solution was dialyzed again with DI water for 5 days, with four changes of the dialysis buffer every day. The resulting chitosan methacrylate solution was lyophilized and stored at −20 °C. ^1^H NMR spectroscopy was used to determine the degree of conjugation of methacrylate to chitosan and was recorded on a Bruker NMR spectrometer (ADVANCE III, 400 MHz) dissolved in D_2_O. The substitution degree (SD) of CMA was determined from the ratio of the integrated area of the Hb-Hf peaks to that of the methylene (Hg) peaks, according to Equation (1) below [11].

(1) $$SD=\frac{\frac{Area\;of\;Peak\;g}{2}}{\frac{Area\;of\;Peak\;b,\;c,\;d,\;e,\;f,}{5}}\times 85\%$$

#### 4.2.2. Synthesis of CMATPP NLG

The CMATPP NLG was fabricated using the ionic gelation method established by Calvo et al. [48] and demonstrated in other studies [52,70], with some changes. CMA was first dissolved in an acidic solvent (0.1 M HCl with 0.9%NaCl), together with LAP to a final concentration of 1 mg/mL, and the probe was sonicated until full dissolution. The CMA solution was stirred at 700 rpm. TPP was dissolved in DI water and filtered to obtain the final concentration of the desired mass ratio with a CMA of (1:7). The TPP solution was injected into the stirred CMA solution, where siRNA was added together with TPP for drug encapsulation and release studies. The resulting nanoparticle solution was then incubated at room temperature for 30 min. After incubation, the nanoparticles were purified by centrifugation at 16,000× *g* for 30 min at 15 °C. The supernatant was discarded, and the pellet was resuspended in an acidic solvent to obtain the nanoparticle solution.

#### 4.2.3. RBC Ghost Membrane

Extraction of RBC membrane ghosts from porcine blood was performed within 4 h of withdrawal from sacrificed swine. The extraction process was adapted from the literature [12,15] and adjusted for suitability with porcine blood. Briefly, porcine blood was centrifuged at 3000× *g* for 10 min at 4 °C to separate RBC from the plasma. The RBC layer was retained and washed thrice with ice-cold PBS, followed by centrifugation at 3000× *g* for 5 min at 4 °C. The washed RBC were resuspended in 0.25× PBS for hypotonic treatment with constant stirring. The burst RBCs were purified by centrifugation at 9000× *g* for 15 min at 4 °C. The pellet was resuspended in hypotonic buffer and washed twice until no red or pink coloration was observed. The final pellet containing RBC membranes was resuspended and stored in a sucrose solution containing 0.35 M sucrose and 1.0 mM EDTA dissolved in PBS (pH 7.4) and stored at −80 °C.

#### 4.2.4. Cell-Derived Nanovesicles

**Figure 9:**
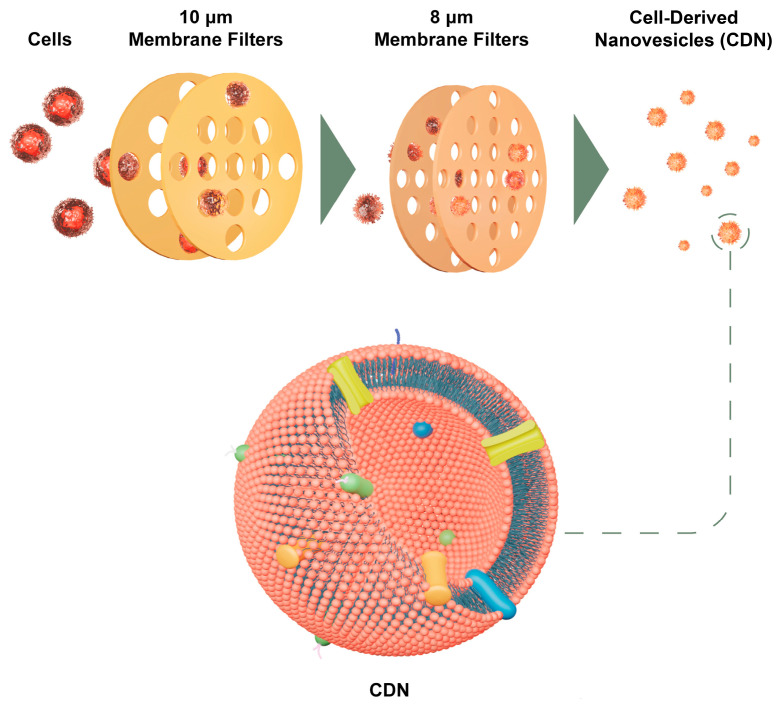
Overall schematic of CDN fabrication using a cell-shearing approach using U937 monocytes.

Cell-derived nanovesicles (CDNs) were prepared as follows [27]. U937 cells were cultured to 80% confluence before centrifugation, washed in PBS, and reconstituted to 1 × 10^7^ cells/mL in PBS. The cell suspension was then added to Pierce™ spin cups (Thermo Fisher Scientific, Singapore) with an attached 10 μm pore size, paper filters, and centrifuged at 14,000× *g* for 10 min at 4 °C. Centrifugation was performed twice through a 10 µm pore filter and twice more through an 8 µm one. The overall schematic is shown below in Figure 9. The filtrate from the final centrifuge was separated by size-exclusion column chromatography using Sephadex G50 (Sigma-Aldrich, St. Louis, MO, USA) soaked in PBS.

#### 4.2.5. Coating of CMATPP NLG

CMATPP was coated with EPC, RBC ghost vesicles, and CDNs to form EPC CMATPP NLG, RBC CMATPP NLG, and CDN CMATPP NLG, respectively. The CMATPP nanoparticles were coated with EPC using a thin-film hydration method [71]. Briefly egg PC was dissolved in chloroform with cholesterol at a 7:3 molar ratio and added to a round-bottom flask. The mixture was spun at 150 rpm for 30 min at 40 °C on a rotary evaporator to dry the lipids. The thin film obtained was hydrated with the CMATPP nanoparticle solution for 30 min at 150 rpm. The resulting multilamellar vesicle (MLV) solution was then extruded through polycarbonate membranes of 400 nm and 200 nm. The obtained particles were crosslinked via photopolymerization under UV light (365 nm) (VL-8. L, Vilber, 1–2 mW/cm^2^) for 5 min to obtain EPC CMATPP NLGs.

The CMATPP nanoparticles were coated with RBC vesicles via co-extrusion. Extracted ghost RBCs were serially extruded through polycarbonate membranes of 400 nm and 200 nm until RBC vesicles of sizes of approximately 200 nm were obtained. The extruded RBC vesicles were then mixed with previously fabricated CMATPP nanoparticles and extruded together through 200 nm polycarbonate membranes to form RBC CMATPP nanoparticles. An overview of this procedure is shown in Figure 2b. The obtained particles were crosslinked via photopolymerization under UV light (365 nm) for 5 min to obtain RBC CMATPP NLGs. CDN coating was performed in the same way, using U937 cells for co-extrusion instead of RBC serially extruded through polycarbonate membranes of 10 μm, 1 μm, and 400 nm.

#### 4.2.6. Microfluidic Coating

**Figure 10:**
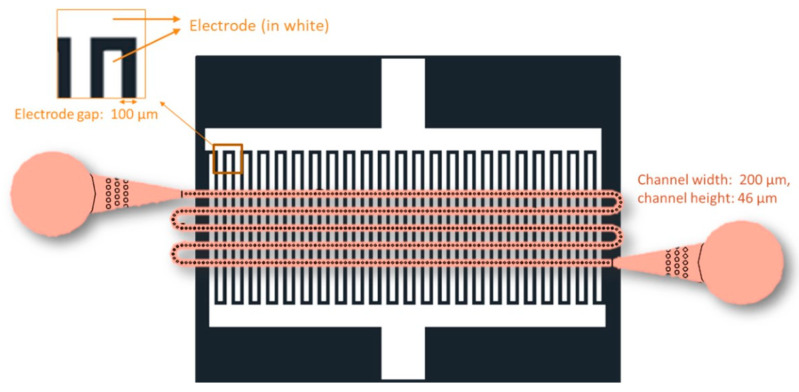
Schematic of the channel design on the microfluidic chip, where the inlet is on the left and outlet is on the right.

The CMATPP was coated with U937 CDNs using a novel microfluidic electroporation device. The chip consisted of a single microchannel channel (200 µm width, 46 µm height) bonded to a glass substrate patterned with microfabricated interdigitated gold electrodes (100 µm width and gap) (Figure 10).

Standard soft lithography was used to fabricate a polydimethylsiloxane (PDMS) channel as previously reported [72]. Briefly, PDMS mixture (10:1, ratio of base elastomer to curing agent) was poured on a SU8-patterend silicon wafer and baked for 1 h at 75 °C. After curing, the PDMS chip was demolded from the wafer, and a 1.5 mm biopsy punch was used to create the inlet and outlet holes. For electrode fabrication, 20 nm thick chromium was sputtered on a quartz wafer before being sputtered with 180 nm gold. The sputtered wafer was diced and plasma-treated for 1 min at 30 W power, with air as the process gas (PDC-002, Harrick Plasma Inc., Ithaca, NY, USA) before bonding with the PDMS channel. The wires were connected to the positive/negative terminals of the sputtered electrode using conductive epoxy (8331D; MG Chemicals, Burlington, ON, Canada). For electroporation, a sample mixture of CDN and CMATPP nanoparticles was perfused into the channel using a syringe pump (CX Fusion 200, Chemyx Inc., Stafford, TX, USA) between 10 and 20 µL/min, followed by electroporation using a square wave between 10 Hz and 1 MHz with a 10% duty cycle generated using a function generator (AFG3102, Tektronix Inc., Beaverton, OR, USA). Owing to aggregation from the electrostatic attraction between the negatively charged CDNs and positively charged CMATPP, the CMATPP and CDN mixture was centrifuged to remove potential aggregates and diluted prior to perfusion into the microfluidic chip. After electroporation, the samples were centrifuged again at 16,000× *g* to remove excess CDNs from the supernatant.

### 4.3. Characterization of CMATPP Nanoparticles

#### 4.3.1. Hydrodynamic Size, Zeta Potential, and Particle Concentration

A Malvern Zetasizer (Nano-ZS90, Malvern Panalytical, Malvern, UK) was used to measure the hydrodynamic diameter, polydispersity indices, and zeta potential of CMATPP nanoparticles. The samples were diluted 75× in distilled water before dynamic light scattering (DLS) measurements using a Zetasizer. Nanoparticle tracking analysis (NTA) was performed to measure the particle concentrations of the samples (Nanosight NS300, Malvern Panalytical, Malvern, UK). The samples were first diluted to the recommended particle concentration per frame and injected into the Nanosight, where an sCMOS camera captured a video of the sample at 25 fps.

#### 4.3.2. Morphology of Nanoparticles

Transmission electron microscopy (TEM) was used to image the CMATPP sample morphologies. In total, 3 µL of samples were pipetted onto TEM copper grids, which had a Lacey Formvar film enforced by a carbon coating and glow discharged, negatively stained with a 2% uranyl acetate solution, and left to dry overnight. The samples were then imaged at 20,000–40,000 times magnification at 120 keV using a JEOL 2010 HR (JEOL, Ltd., Tokyo, Japan) electron microscope. Cryogenic TEM was employed to image the EPC liposomes, during which the samples were plunged frozen in liquid ethane and maintained at liquid nitrogen temperature. Cryogenic TEM imaging was performed using a Carl Zeiss LIBRA^®^ 120 PLUS (Zeiss AG, Oberkochen, Germany) at 120 keV and 80,000–100,000 times magnification.

#### 4.3.3. Flow Cytometry Studies

To confirm that the RBC membrane had coated the UV-crosslinked CMATPP core, samples and controls were treated with Alexa Fluor^®^ 488-conjugated anti-CD47 antibody (Santa Cruz Biotechnology, Inc., Dallas, TX, USA) before flow cytometry was performed on an LSRFortessa™ X-20 Cell Analyzer (BD Biosciences, Franklin Lakes, NJ, USA) and the analysis was performed using FlowJo™ v10.6.1 (BD Biosciences, Franklin Lakes, NJ, USA) software. The samples were prepared by centrifugation at 16,000× *g* for 10 min before the supernatant was discarded, and the pellet was resuspended in PBS (pH 7.4) to deplete loose membrane vesicles and replace the acidic solvent with one under physiological conditions. The antibody was added at 1:200 (*v*/*v*), and the mixture was incubated at room temperature for 1 h. The samples were then centrifuged before the supernatant was discarded, the pellet was resuspended in PBS (pH7.4) as a wash to deplete free antibodies, and flow cytometry was performed.

#### 4.3.4. Drug Loading and Release

One of the most important factors when using a nanoparticulate delivery system is encapsulation efficiency, which is the fraction of total drug used during fabrication that is eventually encapsulated within the CMATPP samples, as shown in Equation (2) below:

(2) $$Encapsulation\;Efficieny\left(\%\right)=\frac{Amount\;of\;Drug\;Encapsulated}{Total\;Amount\;of\;Drug\;Used}\times 100.$$

Common solvents used to break the lipid bilayer, such as ethanol and Triton-X100, affect the sensitivity of the RIBO green assay; hence, the amount of drug encapsulated was calculated from the unencapsulated siRNA in the supernatant after purification of NLG samples and quantified using the Quant-it™ RiboGreen RNA Assay Kit (Thermo Fisher Scientific, Singapore), according to the accompanying standard protocol. siRNA release was monitored for 14 days. The samples were placed in a Spectra/Por^®^ cellulose ester dialysis bag (100 kD, MWCO) (Thermo Fisher Scientific, Singapore) and suspended in 40 times the volume of PBS with 0.05% sodium azide for sink conditions. The release studies were performed at 37 °C with shaking at 100 rpm. At each time point, 1 mL of release buffer was extracted to determine the amount of siRNA released using the Quant-it™ RiboGreen RNA Assay Kit.

### 4.4. Statistical Analysis

Analysis of variance (ANOVA) was conducted using OriginPro 2018 to determine the statistical significance of the mobile fractions for all PEGDA MW, followed by Bonferroni multiple comparisons test. Significance was defined as a *p* value less than 0.05 (* *p* < 0.05).

## 5. Conclusions

In this study, new hybrid CMATPP nanoparticles, fabricated through ionic crosslinking between chitosan methacrylate (CMA) and tripolyphosphate (TPP) followed by a photocrosslinking step for structural stabilization, offer enhanced control over the release profiles of encapsulated biomolecules. This approach overcomes the burst release often seen in traditional delivery systems and introduces the possibility of coating these soft nanoparticles with cellular membranes or extracellular vesicles, expanding their potential applications in targeted and sustained biomolecule delivery.

In general, CMATPP NLGs can be coated through co-extrusion and microfluidics, especially with extracellular vesicles, showing promise for increasing the throughput and automation of the coating process. EVs can be isolated from various cell sources, allowing CMATPP NLGs to acquire diverse membrane properties from different cell types. Potentially, this technology can be developed into customizable, patient-specific therapy where the patient’s cells can be harvested to be used for coating of RNA-loaded CMATPP nanoparticles. This may reduce immunogenicity and improve acceptance of the membrane-coated CMATPP nanoparticles. Nonetheless, more work is still needed to be done to upscale the fabrication of CMATPP nanoparticles, as CMATPP’s stability in neutral pH is dependent on the photocrosslinking of CMA to form covalent bonds, and the use of photoinitiator in that process runs the risk of toxic contaminants from unused photoinitiators and cytotoxicity from exposure to reactive oxygen species [73,74].

## Figures and Tables

**Table 1:** Characterization of CMATPP nanoparticles, bare EPC liposomes, EPC CMATPP NLGs, RBC ghost vesicles, RBC CMATPP NLGs, U937 CDNs, and co-extruded U937 CDN CMATPP NLGs (*n* = 3). Values are presented as (mean ± SD).

Particle Type	CMATPP	Bare EPC Liposome	EPC-CMATPP	Bare RBC Ghosts	RBC-CMATPP	Bare U937 Vesicles	U937-CMATPP
Size (nm)	176.2 ± 3.6	160.5 ± 2.8	185.6 ± 5.2	186.4 ± 4.2	195.3 ± 5.2	216.9 ± 2.8	211.8 ± 3.6
PDI	0.222 ± 0.003	0.085 ± 0.006	0.168 ± 0.021	0.176 ± 0.066	0.303 ± 0.025	0.312 ± 0.015	0.286 ± 0.022
Zeta Potential (mV)	38.2 ± 4.1	−5.2 ± 1.2	7.9 ± 1.3	−29.0 ± 3.2	−12.1 ± 2.4	−35.3 ± 2.4	−28.5 ± 3.9

**Table 2:** Hydrodynamic sizes, zeta potential, and particle concentration data of CMA TPP nanoparticles and U937 CDNs before electroporation and of U937 CDN CMATPP NLGs after electroporation (*n* = 3). Values are presented as (mean ± SD).

Particle Type	CMATPP	Bare U937 Vesicles	U937-CMATPP
Size (nm)	249.7 ± 3.3	198.4 ± 4.5	349.5 ± 5.9
PDI	0.170 ± 0.021	0.399 ± 0.011	0.286 ± 0.022
Zeta Potential (mV)	34.8 ± 2.7	−17.4 ± 3.2	−34.9 ± 4.6
Particle Concentration (Counts/mL)	(1.15 ± 0.11) × 10^11^	(6.25 ± 0.62) × 10^11^	(4.31 ± 0.87) × 10^9^

## Data Availability

All data needed to support the conclusions in the paper are presented in the manuscript and/or the Supplementary Materials. Additional data related to this paper may be requested from the corresponding author upon request.

## Supplementary Materials

The following supporting information can be downloaded at https://www.mdpi.com/article/10.3390/ijms252413724/s1.
