# Supplementary material for: Cell Membrane- and Extracellular Vesicle-Coated Chitosan Methacrylate-Tripolyphosphate Nanoparticles for RNA Delivery
Source: Int J Mol Sci. 2024 Dec 23;25(24):13724. doi: 10.3390/ijms252413724 (PMC11678704; doi:10.3390/ijms252413724)
Supplement: Supplementary file 1 [file ijms-25-13724-s001.zip › ijms-3346484-supplementary.pdf]

International Journal of
Molecular Sciences

Supplementary Material

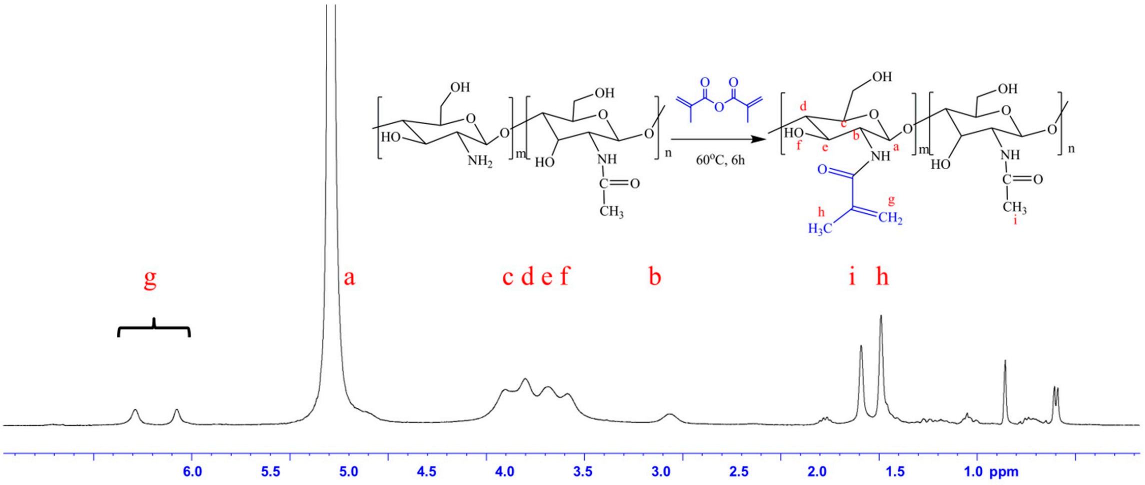

Figure S1 NMR data for CMA

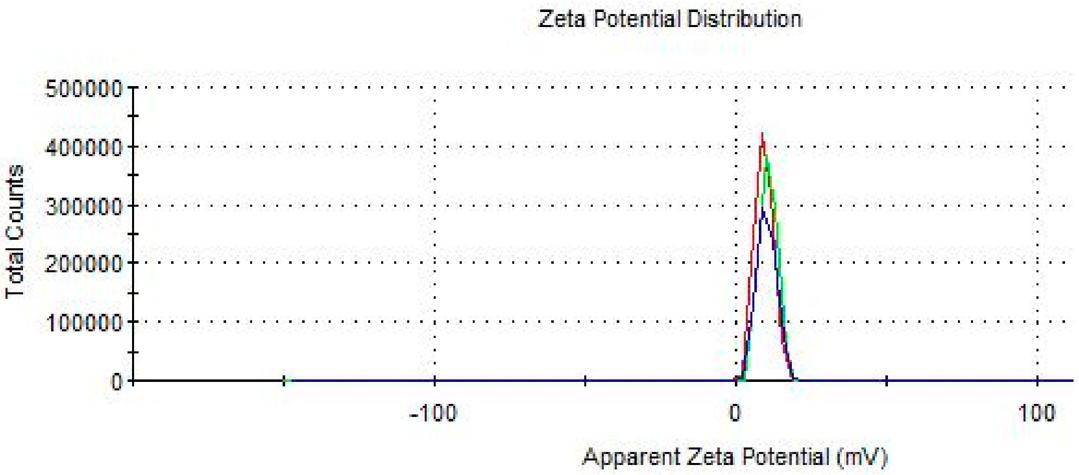

Figure S2 Zeta Potential distribution graph for zeta potential measurements

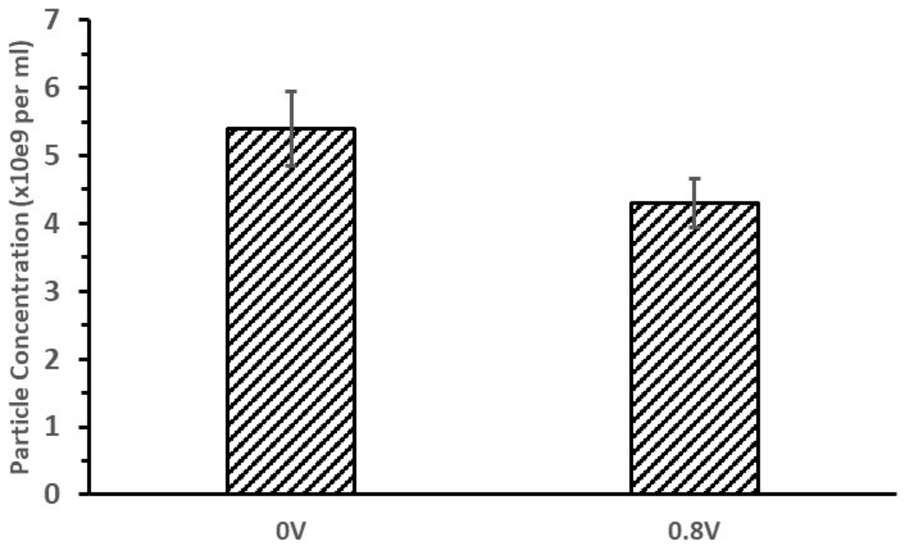

Figure S3 Particle concentration of CDN CMATPP at 0V and 0.8V electroporation. (*p < 0.05)

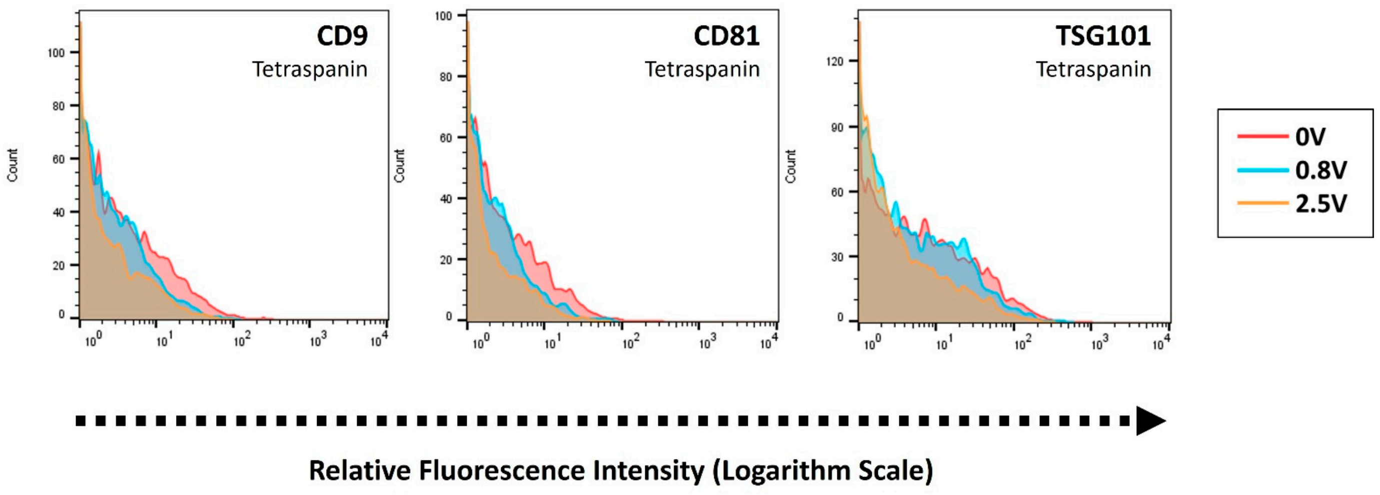

Figure S4 Flow cytometry of three CDN characteristic protein markers' (Tetraspanins: CD9 and CD 81, MVB: TSG101) distribution in CDNs, electroporated under different conditions. Red line depicts electroporation done at 0V, blue line depicts electroporation done at low frequency (100Hz, 0.8V, 1ms pulse width) and yellow line depicts electroporation done at high frequency (1000Hz, 2.5V, 10μs pulse width).

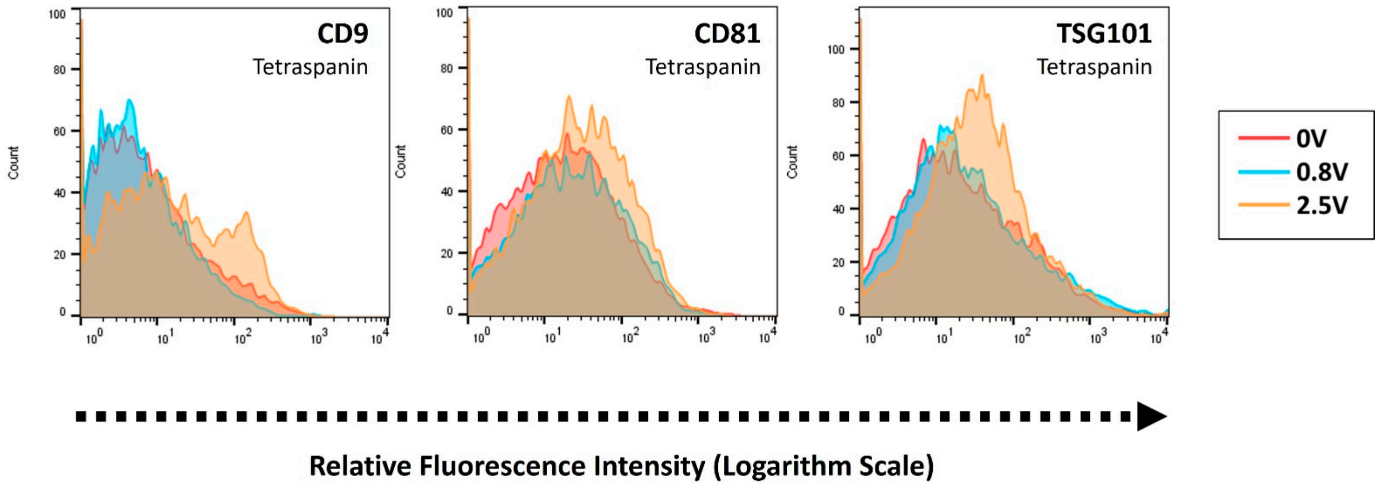

Figure S5 Flow cytometry of three CDN characteristic protein markers' (Tetraspanins: CD9 and CD 81, MVB: TSG101) distribution in CMATPP nanoparticles, electroporated under different conditions. Red line depicts electroporation done at 0V, blue line depicts electroporation done at low frequency (100Hz, 0.8V, 1ms pulse width) and yellow line depicts electroporation done at high frequency (1000Hz, 2.5V, 10μs pulse width).

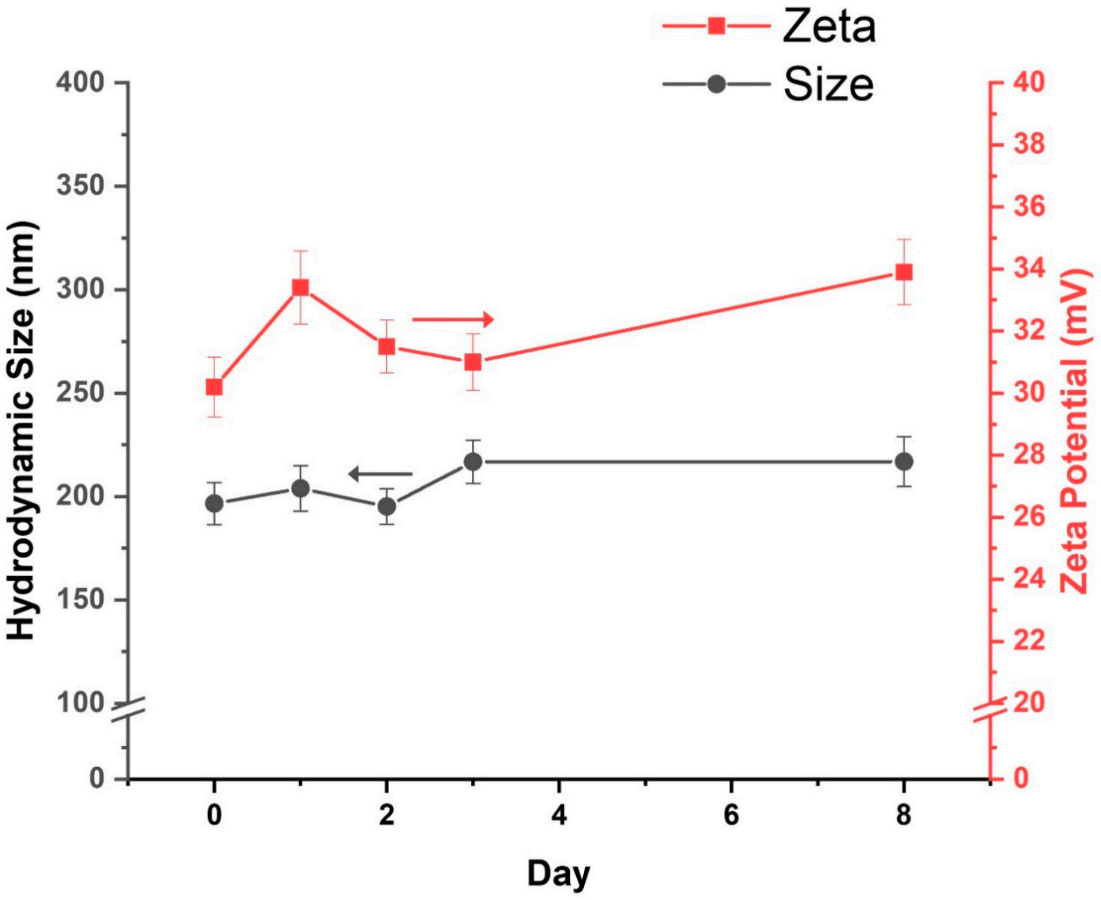

Figure S6 Zeta Potential (Red, square) and hydrodynamic size (Black, circle) data of crosslinked CMATPP nanoparticles in PBS.
